# Inward rectifier potassium (K_ir_) current in dopaminergic periglomerular neurons of the mouse olfactory bulb

**DOI:** 10.3389/fncel.2014.00223

**Published:** 2014-08-08

**Authors:** Mirta Borin, Alex Fogli Iseppe, Angela Pignatelli, Ottorino Belluzzi

**Affiliations:** Department of Life Sciences and Biotechnology, University of FerraraFerrara, Italy

**Keywords:** olfactory bulb, dopaminergic neurons, periglomerular cell, K_ir_ channels, patch-clamp techniques

## Abstract

Dopaminergic (DA) periglomerular (PG) neurons are critically placed at the entry of the bulbar circuitry, directly in contact with both the terminals of olfactory sensory neurons and the apical dendrites of projection neurons; they are autorhythmic and are the target of numerous terminals releasing a variety of neurotransmitters. Despite the centrality of their position, suggesting a critical role in the sensory processing, their properties -and consequently their function- remain elusive. The current mediated by inward rectifier potassium (K_ir_) channels in DA-PG cells was recorded by adopting the perforated-patch configuration in thin slices; I_Kir_ could be distinguished from the hyperpolarization-activated current (I_*h*_) by showing full activation in <10 ms, no inactivation, suppression by Ba^2+^ in a typical voltage-dependent manner (IC_50_ 208 μM) and reversal potential nearly coincident with E_K_. Ba^2+^ (2 mM) induces a large depolarization of DA-PG cells, paralleled by an increase of the input resistance, leading to a block of the spontaneous activity, but the K_ir_ current is not an essential component of the pacemaker machinery. The K_ir_ current is negatively modulated by intracellular cAMP, as shown by a decrease of its amplitude induced by forskolin or 8Br-cAMP. We have also tested the neuromodulatory effects of the activation of several metabotropic receptors known to be present on these cells, showing that the current can be modulated by a multiplicity of pathways, whose activation in some case increases the amplitude of the current, as can be observed with agonists of D2, muscarinic, and GABA_A_ receptors, whereas in other cases has the opposite effect, as it can be observed with agonists of α1 noradrenergic, 5-HT and histamine receptors. These characteristics of the K_ir_ currents provide the basis for an unexpected plasticity of DA-PG cell function, making them potentially capable to reconfigure the bulbar network to allow a better flexibility.

## Introduction

The background potassium conductance mediated by inward rectifying potassium channels impacts on many physiological processes, from the excitability profile of nerve and muscle cells to hormone release. A voltage-dependent block of the channel pore by polyamines and intracellular magnesium is thought to be responsible for the inward rectification of these channels (Lopatin et al., 1995); for a review see Hibino et al. (2010) which, opening at potentials close to E_K_, tend to maintain the membrane in a hyperpolarized state.

Dopaminergic (DA) neurons represent an estimated 10–16% of the neurons residing in the most external (glomerular) layer of the main olfactory bulb (MOB) (Halász et al., 1977; McLean and Shipley, 1988). The glomerular layer (GL) region is occupied by three main types of interneurons, periglomerular (PG) cells, short-axon cells and external tufted (ET) cells - sometimes collectively referred to as juxtaglomerular cells (Kratskin and Belluzzi, 2003; Panzanelli et al., 2007). Dopaminergic neurons in the GL include PG cells (Kosaka et al., 1985; Gall et al., 1987) and a fraction of ET cells (Halász, 1990).

Analyzing the excitability profile of DA-PG cells, we observed that Ba^2+^ (300 μM, a blocker of the K_ir_ channels), induced a large depolarization in bulbar DA neurons, large enough to lead to complete blockage of spontaneous firing of these cells. We then examined the problem, finding that actually there is a K_ir_ current in these cells which escaped a previous investigation of ours (Pignatelli et al., 2005) for its relatively small amplitude in standard saline. The current can be better evidenced with ionic manipulations causing a depolarizing shift of the potassium equilibrium potential, but even under physiological conditions, for the elevated input resistance of these cells, the K_ir_ current is sufficiently large to exert a relevant influence on the cell excitability profile.

Being selective for potassium ions, the channels of the inwardly rectifying family conduct currents which are inward at potentials negative to the K^+^ equilibrium potential (*E*_K_) and outward at potentials positive to *E*_K_, in so doing contributing to the resting membrane potential (Hibino et al., 2010).

## Materials and methods

### Ethic statement

A total of 123 mice have been used. The experimental process was designed so as to minimize animal number and suffering of the animals used. The protocols adopted were designed according to European Council Directives (609/1986 and 63/2010) and Italian laws (DL 116/92) on the protection of animals used for scientific purposes. The experimental procedures were approved by the Ethical Committee for Animal Experiments of the Ferrara University (CEASA), by the Directorate-General for Animal Health of the Ministry of Health, and supervised by the Campus Veterinarian of the University of Ferrara.

### Animals and surgical procedures

For these experiments we used a transgenic mice strain (TH-GFP/21–31), carrying the eGFP transgene under the control of the TH promoter (Sawamoto et al., 2001; Matsushita et al., 2002). The TH-GFP strain was maintained as heterozygous by breeding with C57BL/6J inbred mice.

### Recording conditions

The temperature of the 1-ml recording chamber was controlled using Peltier devices (RS Components, Milan, Italy) and measured with a high-precision, type K thermocouple (RS Components).

For current and voltage recordings an Axopatch 200B amplifier (Molecular Devices, Sunnyvale, CA) was used, and the signals were digitized and acquired with a Digidata 1440A (Molecular Devices) 16 bit A/D–D/A converter; correction for the junction potential was calculated using the related function of the acquisition software (pClamp 10, Molecular Devices).

Patch pipettes were built from borosilicate glass capillaries (1.5 o.d., 0.87 i.d., with filament; Hilgenberg, Malsfeld, Germany) with a Zeitz-DMZ puller (Martinsried, Germany), and showed a resistance of 4–5 MΩ when filled with standard intracellular solution (see below); the seal formation was assisted by a MCPU-3 air pressure controller (MPI, Lorenz Meβ gerätebau, Katlenburg-Lindau, Germany); the seal resistance obtained was always greater than 3 GΩ.

### Solutions

The solutions used had the following composition (mM):

- *EC0*, standard ACSF extracellular (EC) solution: 125 NaCl, 2.5 KCl, 26 NaHCO_3_, 1.25 NaH_2_PO_4_, 2 CaCl_2_, 1 MgCl_2_, and 15 glucose.
- *EC1*, high K EC solution: 95 NaCl, 32.5 KCl, 26 NaHCO_3_, 1.25 NaH_2_PO_4_, 2 CaCl_2_, 1 MgCl_2_, and 15 glucose.
- *EC2*, K-TEA EC solution: 100 NaCl, 2.5 KCl, 26 NaHCO_3_, 1.25 NaH_2_PO_4_, 2 CaCl_2_, 1 MgCl_2_, 20 TEA, and 10 glucose.
- *EC3*, high K-TEA EC solution: 70 NaCl, 32.5 KCl, 26 NaHCO_3_, 1.25 NaH_2_PO_4_, 2 CaCl_2_, 1 MgCl_2_, 20 TEA, and glucose.

All EC solutions were continuously bubbled with 95% O_2_ and 5% CO_2_, and the osmolarity was corrected to 305 mOsm with glucose.

*Standard pipette-filling intracellular (IC) solution*: 120 KCl, 10 NaCl, 2 MgCl_2_, 0.5 CaCl_2_, 5 EGTA, 10 HEPES, 2 Na-ATP, 10 glucose; in this solution, the free IC calcium concentration was calculated to be 16 nM (http://www.stanford.edu/~cpatton/downloads.htm).

For perforated patches, 200 μg/ml amphotericin B was added to the IC solution (plus 300 μg/ml pluronic F-127). EGTA was omitted and CaCl_2_ concentration was increased to 3 mM in the electrode filling solution to control of the integrity of the perforated patch, as in case of unexpected rupture, the massive entry of calcium from the pipette would cause a rapid cell death. Data were collected after the series resistance dropped below 50 MΩ.

In all IC solutions the osmolarity was finely tuned to 295 mOsm with glucose, and the pH to 7.2 with KOH.

Except where indicated, when recording from slices, the EC solutions included two mixtures of blockers:

- *BL1*, for ligand-gated channels (1 mM kynurenic acid and 10 μM bicuculline).
- *BL2*, for voltage-dependent channels (TTX 0.6 μM, Cd^2+^ 100 μM and ivabradine 10 μM).

### Analysis of current recordings

I_Kir_ amplitude was measured as instantaneous current at the beginning (I_inst_) and at the end of test voltage pulses as steady-state current (I_ss_).

The temperature-dependence of activation and deactivation rate constants were calculated as:

(1) $$Q_{10}\;=\;{\left(\frac{rate\left(T_{2}\right)}{rate\left(T_{1}\right)}\right)}^{\frac{10}{T_{2}-T_{1}}}$$

where *Q*_10_ is the fold-change as a consequence of increasing the temperature by 10°C, calculated between the two temperatures T_1_ and T_2_.

### Data analysis

To evoke the K_ir_ current, a series of hyperpolarizing voltage steps in −10 mV increments were imposed from the holding potential of −40 to −130 mV at 10 s intervals. Unless otherwise indicated, the current amplitudes were measured at the end of the hyperpolarizing step (steady-state current).

When box charts are used to represent data ensembles, the central square represents the mean, the central line the median, the range of the boxes represent the S.E, and the whiskers define the 10–90% range of data samples.

Offline analysis was performed using version 10.3 of pClamp (Molecular Devices) and version 8.1 of OriginPro (OriginLab Corporation, Northampton, MA).

Unless otherwise indicated, data are presented as means ± s.e.m.; for the statistical analysis we used the software Prism 5 (GraphPad, La Jolla, CA). The statistical significance was assessed with Two-Way analysis of variance (ANOVA), or Student's *t*-test for paired samples as indicated; in Two-Way ANOVA multiple comparisons post-tests were performed using the Bonferroni method.

*P* value of < 0.05 was considered significant; in figures, 1 to 4 asterisks represent differences significant at the 0.05, 0.01, 0.001, 0.0001 level, respectively.

## Results

The data are based on recordings from 285 TH+ PG neurons from the glomerular layer. Most OB DA cells are small, PG interneurons (about 5–8 μm in diameter), but there is also a certain number of external tufted (ET) cells (about 10–15 μm in diameter) (Baker et al., 1993; Kosaka and Kosaka, 2009, 2011). In this study, we restricted the analysis to PG cells; these were selected on the basis of their location around the glomerular border, dendritic arborization extending within the glomerular neuropil, membrane capacitance (8.0 ± 0.2 pF; *n* = 297) and input resistance (979.4 ± 33.4 MΩ; *n* = 276). In addition to the evident differences in dimension (Kosaka and Kosaka, 2008), membrane capacity and input resistance (Pignatelli et al., 2005), DA-PG cells show a regular firing pattern, whereas DA-ET cells show burst pattern activity (Hayar et al., 2004). Finally, short-axon cells, which have membrane capacitance and input resistance very similar to PG cells, can be usually recognized in slice for their fusiform shape, position amid different glomeruli, and dendrites extending between neighboring glomeruli (Shipley and Ennis, 1996).

### Identification and basic properties of the current

**Figure 1:**
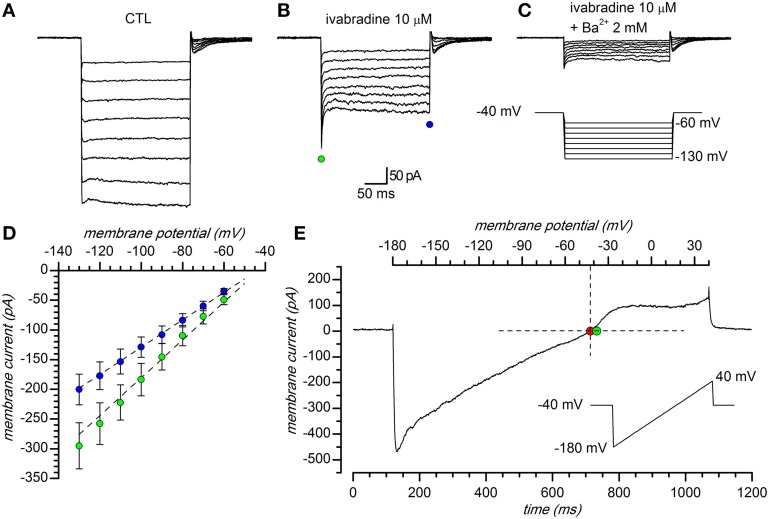
**General properties. (A–C)** Representative currents obtained in response to hyperpolarizing pulses in 32.5 mM external K^+^ solution: **(A)** EC1 saline with 1 mM kynurenic acid, 10 μM bicuculline (BL1 mix), 0.6 μM TTX, 100 μM Cd^2+^; **(B)** same solution as in A plus 10 μM ivabradine; **(C)** same solution as in B plus 2 mM Ba^2+^. Voltage steps from a holding potential of −40 mV with hyperpolarizing steps ranging from −60 to −130 mV in 10 mV increments. **(D)**: I–V relationship of peak (green dots) and steady-state (blue dots) current; mean current amplitude of 81 cell recordings. Vertical error bars represent standard error; EC2 saline, with BL1 and BL2 mixes of blockers. **(E)** Instantaneous I/V curve during application of a 220 mV/s ramp protocol (from −180 to +40 mV, 0.23 V/s) in a DA PG cell perfused with the solution described in **(A)**, after subtraction of the ohmic leak; the red dot (−41.3 mV) marks the observed reversal potential, the green dot the Nernstian equilibrium potential in the experimental conditions used ([K^+^]_o_ = 32.5 mM). All the experiments shown in this figure were performed in slice, perforated patch configuration, at 34°C.

In a first series of experiments, carried out using perforated patch recordings in slice at 34°C, hyperpolarizing steps were delivered from a holding potential of −40 mV, which is close to the predicted K^+^ equilibrium potential in our experimental conditions (E_K_ = −37.7 mV for [K^+^]_o_ = 32.5 mM), to potentials ranging from −60 to −130 mV (10 mV increments, 10 s interval). The inward current obtained in response is shown in Figure 1A; a fraction of this current could be suppressed by two organic compounds known as selective HCN channels blockers, i.e., ZD7288 30 μM (BoSmith et al., 1993) and S-16257, a.k.a. ivabradine, 10 μM; (Bois et al., 1996; Bucchi et al., 2002), Figure 1B; the h-current in DA-PG cells has been the object of another study (Pignatelli et al., 2013), and will be not further discussed in this paper.

The current activated by hyperpolarization remaining after suppression of the h-current, was suppressed by Ba^2+^ (Figure 1C), a classical blocker of K_ir_ channels (Hagiwara et al., 1978; French and Shoukimas, 1985); for its potassium and voltage-dependence, reversal potential and sensitivity to Ba^2+^ this component was identified as potassium inward rectifier (K_ir_) current (Hibino et al., 2010). The I/V relationship of the current evoked by hyperpolarization in a group of 81 cells in the presence of 0.6 μM TTX, 100 μM Cd^2+^ and 10 μM ivabradine is shown in Figure 1D; here, and in the following experiments, for the inherent difficulties, the leakage component of the current evoked by hyperpolarization was not subtracted.

#### Barium sensitivity

**Figure 2:**
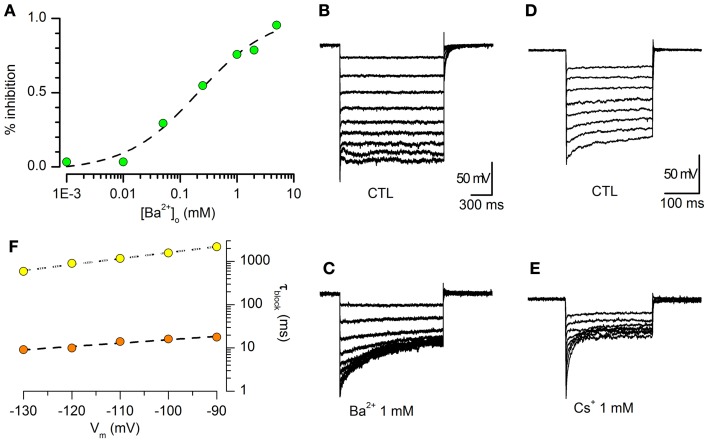
**Barium sensitivity. (A)** Percentage inhibition of steady-state K_ir_ current vs. [Ba^2+^]o. [K^+^]_o_ = 2.5 mM, 32°C. The data (green dots) were fitted with a logistic function (see text), giving a concentration at half-block (Kd) of 0.21 ± 0.10 mM, and a slope (Hill coefficient) of 0.69 ± 0.23 (*n* = 5, −120 mV). **(B,C)**: sample tracings obtained in a single cells in response to hyperpolarizing steps from −40 to −120 mV in standard saline plus BL1 and BL2 **(B)**, and in the presence of 1 mM Ba^2+^ **(C)**. **(D,E)**: same experimental conditions and protocol as above; blocking effect of 1 mM Cs^+^. **(F)** Voltage dependence of the blocking time constant; the data points were obtained in the presence of 1 mM Ba^2+^ (yellow dots) or Cs^+^ (orange dots).

The Ba^2+^ dependent block of I_Kir_ was evaluated from the decrease of steady-state current amplitude at −120 mV in the presence of increasing external Ba^2+^ concentrations. In Figure 2A is represented the percentage of the current inhibition as a function of external Ba^2+^ concentrations ranging from 1 μM to 10 mM. The data could be interpolated by a logistic equation with the form:

(2) $$y\;=\;I_{max}/\;\left(1\;+\;{\left({\left[Ba^{2+}\right]}_{o}\;/K_{d}\right)}^{H}\right)$$

where I_max_ is the asymptotic value of the current block, K_d_ the external Ba^2+^ concentration causing 50% block, and *H* is the slope of the dose-response curve (Hill coefficient). The fit of the Ba^2+^ block of peak I_Kir_ gave a K_d_ of 0.21 ± 0.10 mM and a *H* value of 0.69 ± 0.23 (*n* = 5, −120 mV).

#### Voltage-dependence of the steady-state block by Ba^2+^ and Cs^+^

Ba^2+^ and Cs^+^ have been found to block the K_ir_ channel through an interaction which is thought to occur via a deep binding site, located approximately half-way along the channel (Standen and Stanfield, 1978; Shieh et al., 1998; Alagem et al., 2001). As normally occurs for deep-site blockers, Ba^2+^ and Cs^+^ block is highly voltage dependent (Hagiwara et al., 1978; Harvey and Ten Eick, 1989; Alagem et al., 2001). The effect induced by 1 mM Ba^2+^ in DA-PG cells is shown in Figures 2B,C. The time required for the blocking reaction to reach steady state was calculated by fitting the exponential decay of the currents to the function:

(3) $$I\;=\;A\;exp\text{( }-\;t\text{/}t_{blk}\text{) + }C$$

where A is the current amplitude, *t* is the independent variable, C is the current amplitude at the steady-state, and τ_blk_ is the blocking time constant, whose voltage dependence is shown in Figure 2F.

We also tested the effects of 1 mM Cs^+^, another classical blocker of this channel for which the approach to steady-state block following a voltage step is much faster than for Ba^2+^ (Hagiwara et al., 1976, 1978; Shioya et al., 1993). The results, shown in Figures 2D–F, are in good agreement with those reported in literature (Hagiwara et al., 1976, 1978).

#### Reversal potential

**Figure 3:**
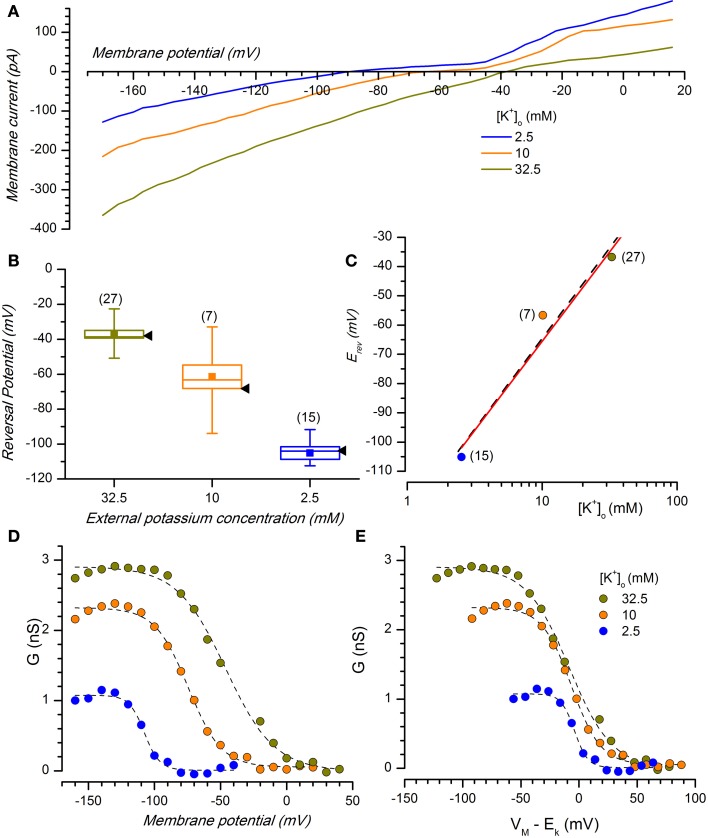
**Potassium sensitivity. (A)** Effect of changing [K^+^]o on membrane current. Average currents (*n* = 8) at the indicated external potassium concentrations in response to voltage ramps from −170 to +20 mV from a holding potential of −40 mV, 0.22 V/s; perforated patches; the bathing solution included Bl1 and Bl2. **(B)** Box charts showing the reversal potentials at different [K^+^]o [same color code as in **(A)**]; black arrow heads to the right of each box mark the expected reversal potentials predicted by the Nernst equation. In the box charts, here and in the following, the square in the center of the box represents the mean value, the horizontal line crossing the box indicates the median, the range of the box represents standard error and the whiskers define the 10–90% range of data sample. **(C)** Plot of the reversal potential for the inwardly rectifying current against the logarithm of [K^+^]_o_. The linear regression fit (black dash line) has a slope of −61.9 mV, close to the theoretical value of −61 mV predicted by the Nernst equation (red line). **(D)** K^+^- and voltage-dependence of chord conductance (g_Kir_); the chord conductance was calculated using the equation g_Kir_ = I_Kir_/(V_m_ − E_K_), where I_Kir_ = steady state current. g_Kir_ plotted as a function of voltage-clamp test potentials at 2.5, 10, and 32.5 mM [K^+^]_o_. **(E)** Data in **(D)** replotted as a function of the driving force. Data points were fitted by Boltzmann curve using a least-squares method; *n* for 2.5, 10, and 32.5 mM was 7, 5, and 12, respectively.

The K_ir_ channels are selective for K^+^ ions, and consequently the reversal potentials of the inward rectifying current for different extracellular K^+^ concentrations should always follow the Nernstian equilibrium potential for potassium (Figures 1E, 3A). When the [K^+^]_o_ was changed from 2.5 to 10 and 32.5 mM, the reversal potentials progressively shifted toward more positive potentials (−105.12 ± 3.67 mV, *n* = 15, for 2.5 mM; −56.67 ± 9.78 mV, *n* = 9, for 10 mM; −36.78 mV ± 1.89, *n* = 27, for 32.5 mM); the reversal potentials in the different experimental conditions are represented in Figure 3B, where they are compared to the theoretical Nernstian equilibrium potentials for K^+^ ions (black triangles). The plot of the reversal potentials against the logarithm of [K^+^]_o_ gives a linear relationship (*r*^2^ = 0.93) with a slope of −61.9 mV, close to the theoretical value of −61.0 mV predicted by the Nernst equation (Figure 3C).

#### K^+^ and voltage dependence of the I_Kir_

Besides the selectivity to K^+^ ions, another typical characteristic of this current is a voltage-dependence of the K_ir_ conductance (g_Kir_) on the K^+^ reversal potential; then, in DA-PG cells we further examined the dependence of g_Kir_ from membrane potential for different external K^+^ concentrations.

The conductance-voltage relationship showed the typical sigmoidal profile, increasing at negative potentials and with a point of half-activation approximately centered at E_K_ (Figure 3D). Plotting the conductance for different [K^+^]_o_ as a function of the driving force (V_m_ − E_K_, Figure 3E), the midpoints were approximately aligned at the zero of the abscissa axis, with minima and maxima at the same voltage levels. This confirms that K_ir_ conductance in DA-PG cells has a voltage-dependence which is function of E_K_, in analogy to what has been found for I_Kir_ in several other preparations (Hestrin, 1981; Leech and Stanfield, 1981; Harvey and Ten Eick, 1988).

#### Effect of I_Kir_ on membrane potential and input resistance

**Figure 4:**
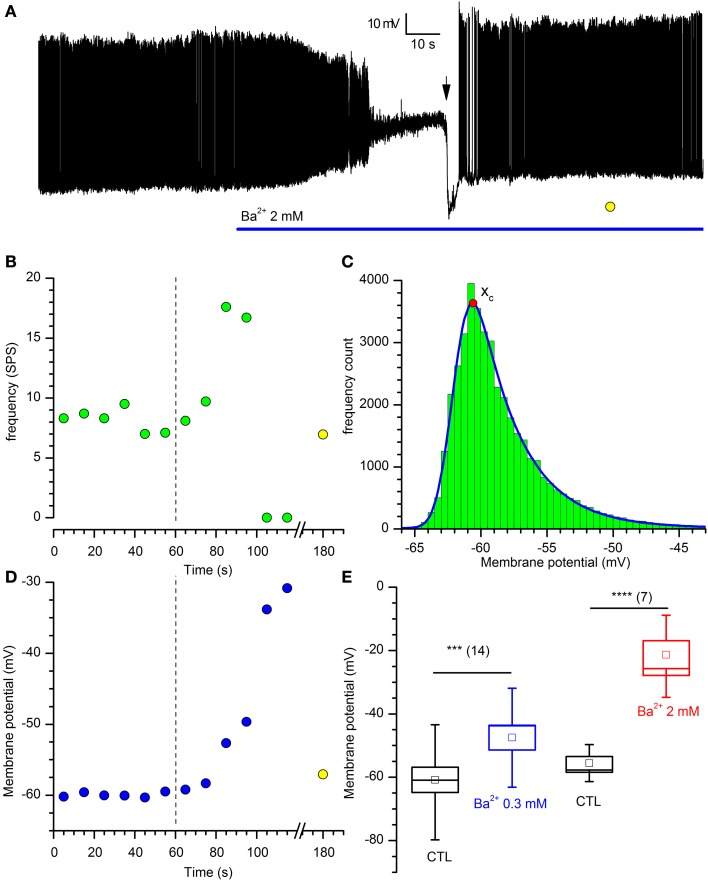
**Effects of Barium on DA-PG cells. (A)** Effect of Ba^2+^ on membrane potential. Perforated patch recording in standard saline (EC1 solution). The blue bar indicates the time of application of 2 mM Ba^2+^ into the bath; starting at the time indicated by the downward arrow, a 40 pA hyperpolarizing current was injected; (further explanation in the text). **(B)** Frequency analysis of action potentials (SPS, spike per second) for the experiment shown in panel A; the dashed line marks the time at which Ba^2+^ has been applied and the yellow point after the x-axis interruption is a measure of the activity after the injection of a hyperpolarizing current, at the time marked by a yellow point in **(A)**. **(C)** Illustration of the method used for the calculation of the prevailing membrane potential (further explanation in the text): 10 s frequency count histograms of the membrane potential were realized at 10 s intervals, and the distributions were fitted by an exponentially modified Gaussian function (equation 3 in the text); the point marked by the red dot indicates the prevailing membrane potential (xc in equation 3). **(D)** Depolarization induced by 2 mM Ba^2+^ in the experiment shown in **(A)** using the analysis of the prevailing membrane potential (blue dots); the dashed line marks the time at which Ba^2+^ has been applied and the yellow point after the x-axis interruption is a measure of the membrane potential at the time marked by a yellow point in **(A)**. **(E)** Depolarization induced by two different concentrations of [Ba^2+^]_o_: 13. 3 ± 2.2 mV for 300 μM (*n* = 14), and 38.1 ± 6.0 mV for 2 mM (*n* = 7).

If the I_Kir_ is active at rest, then it should be expected that the block of the current with Ba^2+^ should influence both input resistance and resting potential; in effect, Ba^2+^ (2 mM) induces a rapid and strong depolarization of DA-PG cells (Figures 4A,C), paralleled by an increase of the firing frequency before its block in depolarization (Figures 4A,B).

The K_ir_ current is not essential to the pacemaker process, as the injection of hyperpolarizing current (40 pA at the time marked by a downward arrow in the representative experiment shown in Figure 4A) resumes completely the activity.

To find a parameter accounting for the “resting” membrane potential in a cell characterized by autorhythmicity, we have calculated the potential at which the cell was staying most of the time, that we have defined “prevailing membrane potential,” using the method illustrated in Figure 4C: frequency count histograms of the digitized membrane potential were obtained at 10 s intervals, and the distributions were fitted by an exponentially modified Gaussian function (Kalambet et al., 2011) with the form:

(4) $$f(x)\;=\;y_{0}\;+\;\frac{A}{t_{0}}e^{\frac{1}{2}{\left(\frac{w}{t_{0}}\right)}^{z}-\frac{x-x_{c}}{t_{0}}}\int_{-\infty }^{z}\frac{1}{\sqrt{2\pi }}e^{-\frac{y^{2}}{2}}dy$$

where

$$z\;=\;\frac{x-x_{c}}{w}\;-\;\frac{w}{t_{0}}$$

and *y*_0_ is the offset, *A* is the amplitude, *x*_c_ is the center of the peak (i.e., the prevailing potential, red dot in Figure 4C), *w* is the width of the peak and *t*_0_ is the modification factor (skewness, *t*_0_ > 0).

Using this method, we measured the variation of the prevailing membrane potential for two different external Ba^2+^ concentrations (0.3 and 2 mM). In a group of cells, we measured a depolarization from −59.1 ± 4.1 to −45.94 ± 4.0 mV with 0.3 mM Ba^2+^ (Figure 4E; *n* = 14, *p* = 0.000025, *t*-test for paired data), and from −52.3 ± 3.7 to −16.2 ± 4.9 mV with 2 mM Ba^2+^ (Figure 4E; *n* = 7, *p* = 0.0006, *t*-test for paired data).

**Figure 5:**
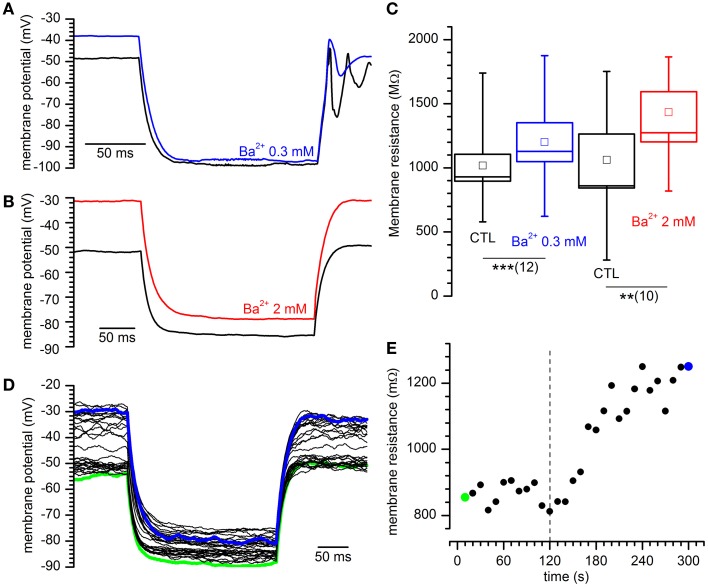
**Effect of different concentrations of Ba^2+^ on input resistance. (A,B)** Sample tracings showing the response to the injection of 40 pA in current-clamp conditions for the indicated external Ba^2+^ concentrations. **(C)** Increase of input resistance at the indicated external Ba^2+^ concentrations: +17.8 ± 3.2%, *n* = 12, and 58.7 ± 14.2%, *n* = 10 in 0.3 and 2 mM external [Ba^2+^]o with respect to controls. ^**^ and ^***^ indicate significance levels of 0.01 and 0.001, respectively. **(D)** Family of tracings obtained in response to hyperpolarizing current pulses as indicated in **(A)**; green and blue traces are taken at the beginning and at the end of a 5′ test; Ba^2+^ was applied after 2′. **(E)** Time course of the variation of input resistance for the experiment shown in **(D)**; the dashed line marks the time of application of Ba^2+^ 2 mM; green and blue dots mark the resistance of the traces with the same color in **(D)**.

Next, we tested the variations of the input resistance in response to hyperpolarizing current pulses in presence of 0.3 and 2 mM Ba^2+^ (Figures 5A,B). In these conditions, for both concentrations we observed an increase of the membrane impedance (Figures 5D,E). In Ba^2+^ 0.3 mM the membrane impedance changes from 1079.6 ± 163.9 to 1260.0 ± 186.5 MΩ (Figure 5C; *n* = 12, *p* = 0.00033, *t*-test for paired data), and in Ba^2+^ 2 mM the mean value changes from 1061.6 ± 202.0 MΩ to 1621.2 ± 284.2 MΩ (*n* = 10; Figure 5C; *p* = 0.0018, *t*-test for paired data).

#### Effect of temperature

**Figure 6:**
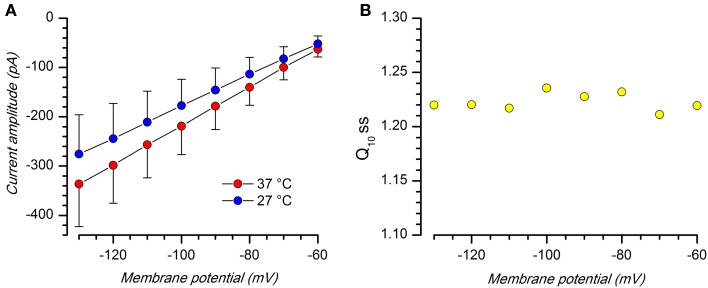
**Effect of temperature. (A)** Comparison of K_ir_ current amplitude (Iss) at 27 and 37°C; EC2 solution with BL1 and BL2; *n* = 8. **(B)** Q10 at the different voltages; the mean value is 1.22 ± 0.003; *n* = 8.

As for the other K currents, also the K_ir_ kinetics is sensitive to thermic conditions. The temperature at which electrophysiological recordings are obtained influence the current kinetics (Figure 6A), and therefore in this study all recordings were made in controlled temperature conditions.

Q_10_ at the different voltages, measured using Equation 1, is substantially stable, with a mean value of 1.22 ± 0.008 (*n* = 9; Figure 6B), a value which is typical for inward rectifying K-conductances (Leech and Stanfield, 1981; Mitsuiye et al., 1997; Paajanen and Vornanen, 2003).

### Pharmacology

#### Blockers

Although the involvement of K_ir_ channels has been demonstrated in numerous common disorders, including hypertension, cardiac arrhythmias and pain, their pharmacology is virtually limited to Ba^2+^, Cs^+^, and few poorly selective cardiovascular and neuroactive drugs with off-target activity toward these channels (Bhave et al., 2010; Hibino et al., 2010; Lüscher and Slesinger, 2010).

***Tertiapin***. Tertiapin, a toxin from the honey bee (*Apis mellifera*), is a remarkable exception, as it is a rather selective blocker of Kir1.1 and Kir3.1 – 3.4 channels (Jin and Lu, 1998; Dobrev et al., 2002; Ramu et al., 2008). The former, renal outer medullary potassium channels, are of no interest in our case, but the latter (G protein-coupled K_ir_, a.k.a. GIRK, channels) are present in the periglomerular layer of the MOB (Karschin et al., 1996), and therefore it was of some importance to test the efficacy of the drug in our cells.

**Figure 7:**
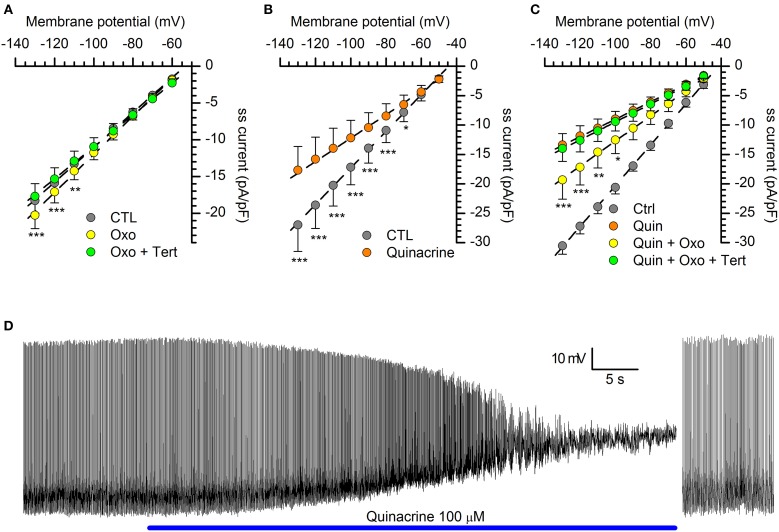
**Organic blockers of the K_ir_ channels. (A)** with a GIRK current activated by a cholinergic muscarinic agonist (oxotremorine 10 μM), the GIRK channels blocker tertiapin-Q (1 μM) completely suppresses the current (*n* = 11; see text for explanation); tertiapin-Q alone does not change the amplitude of hyperpolarization-activated (not shown; *n* = 13). In this as in the following panels, the steady-state (ss) current is calculated in relation to membrane capacity. **(B)** Quinacrine (100 μM) inhibition of hyperpolarization-activated current (*n* = 15). **(C)** With the KIR2.x channels blocked by quinacrine, a muscarinic cholinergic agonist (oxotremorine 10 μM) can activate a GIRK current (yellow dots), and this fraction can be completely suppressed by tertiapin (green dots; *n* = 7). ^*^, ^**^ and ^***^ indicate significance levels of 0.05, 0.01 and 0.001, respectively. **(D)** Effect of quinacrine on membrane potential. Perforated patch recording in standard saline (EC1 solution). The blue bar indicates the time of application of 100 μM into the bath; to the right, a sequence recorded after injection of 35 pA hyperpolarizing current; (further explanation in the text). All recordings were realized at 34°C, in EC2, BL1, and BL2; statistical analysis performed with Two-Way ANOVA and *post-hoc* Bonferroni test.

The oxidation-resistant form of the drug, tertiapin-Q, was ineffective when tested alone at concentrations ranging from 100 nM to 3 μM (not shown). However, GIRK channels become activated only following the binding of ligands to their cognate G protein-coupled receptors, which causes the dissociation of the βγ subunits of a pertussis toxin-sensitive G protein which subsequently bind to and activate the GIRK channel (Walsh, 2011). Therefore, we tested the effect of tertiapin after activation of K_ir_ current with oxotremorine, a metabotropic cholinergic receptor activator (see also below). In these conditions, tertiapin completely abolished the current increment promoted by the muscarinic receptor activation (Figure 7A), suggesting that functional GIRK channels are actually present in DA-PG cells.

***Quinacrine***. Quinacrine is a molecule developed in the 1920s as anti-malarial agent, based upon the aminoacridine ring structure; more recently, it has been shown to inhibit different ionic currents, like the I_A_ (Kehl, 1991), the L-type Ca^2+^ current (Nagano et al., 1996) and the inward rectifier K^+^ current (Evans and Surprenant, 1993; López-Izquierdo et al., 2011). We then tested quinacrine (100 μM), which suppresses a significant fraction of the hyperpolarization-activated current in DA-PG cells (Figure 7B): for voltage commands to -100 mV, the amplitude of the inward current was reduced from −17.16 ± 3.00 pA/pF (CTL) to −12.19 ± 2.98 pA/pF (*p* < 0.001; *n* = 9; Two-Way ANOVA). With the 2.x channels blocked by quinacrine (Figure 7C, orange dots), oxotremorine was still capable of increasing the hyperpolarization-activated current (Figure 7C, yellow dots), increase that could be blocked by tertiapin (Figure 7C, green dots), in agreement with the selectivity of the drug for GIRK channels.

Quinacrine 100 μM was applied in *current-clamp* recordings to verify its capacity to reproduce the barium effect on membrane potential. Quinacrine, which -unlike barium- blocks the K_ir_ current with a voltage-independent mechanism, causes a large depolarization leading to a complete suppression of firing activity (Figure 7D). However, following the injection of hyperpolarizing current bringing the membrane potential back to resting values, the spontaneous activity was resumed (Figure 7D, right), a result confirming that the K_ir_ current exerts a tonic control of the resting potential, but is not an essential component of the pacemaker mechanism.

Quinacrine has been reported to have a psychotic side effect (Lindenmayer and Vargas, 1981), via inhibition of PLA_2_ and increase of DA release (Reid et al., 2002), but we can reasonably exclude this mechanism in our case as DA increases the K_ir_ amplitude (see below).

#### K_ir_ modulation by cAMP

The inward rectifier potassium current can be modulated by cAMP, which has been found to either inhibit (Ito et al., 1997; Jakob and Krieglstein, 1997; Xu et al., 2002; Podda et al., 2010) or enhance the current (Park et al., 2005; Bolton and Butt, 2006) in different preparations.

**Figure 8:**
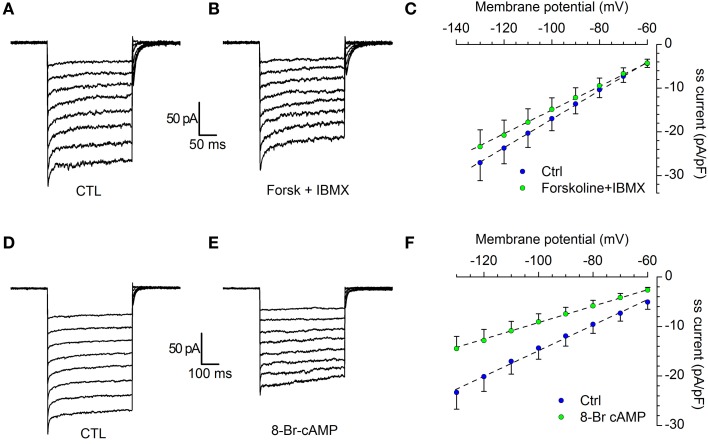
**Modulation of I_Kir_ by cAMP. (A–C)** Effect of forskolin. Current tracings in control **(A)** and in the presence of 10 μM forskolin plus 100 μM IBMX **(B)**; **(C)** comparison of the I/V curves recorded in control (blue dots) and forskolin (green dots); *n* = 12; the difference, tested with Two-Way ANOVA and *post-hoc* Bonferroni test, is significant for the potentials more negative than −80 mV. **(D–F)** Effect of 8Br-cAMP. Current tracings in control **(D)** and in the presence of 10 μM 8Br-cAMP **(E)**; **(F)** Comparison of the I/V curves recorded in control (blue dots) and 8Br-cAMP (green dots); *n* = 6; the difference, tested with Two-Way ANOVA and *post-hoc* Bonferroni test, is significant for the potentials more negative than −80 mV. All recordings were realized in EC2 with the addition of BL1 and BL2, at 34°C.

Under voltage-clamp conditions, the addition to the extracellular solution of 10 μM forskolin, a classical activator of adenylyl cyclase (Seamon and Daly, 1981) and 0.1 mM IBMX, a phosphodiesterase inhibitor, induces a decrease of the K_ir_ current (Figures 8A–C): the stimulation of the cAMP synthesis reduces the I_Kir_ amplitude of 12.3 ± 0.22 % in the range from −80 to −130 mV (*n* = 12; *p* < 0.01).

The experiment was repeated in the same testing conditions, but using 10 μM 8Br-cAMP. The effect was more marked, with a 36.9 ± 0.15 % reduction of current amplitude (Figures 8D–F; *n* = 6; *p* < 0.001). In both cases, the difference among control and test was significant in the range of potentials more negative than −80 mV (Two-Way ANOVA).

#### K_ir_ modulation by neurotransmitters

Dopaminergic cells in the MOB are the target of numerous afferents releasing a variety of neurotransmitters potentially capable of a modulation of the K_ir_-current, including some which are known to affect the cAMP pathway.

**Figure 9:**
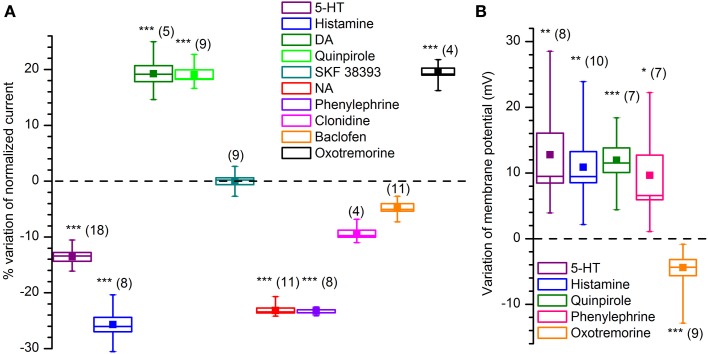
**Effect of various neurotransmitters and agonists acting on I_Kir_. (A)** Effect on current amplitude in voltage-clamp conditions. **(B)** Effect on membrane potential in current-clamp conditions. All recordings were realized at 34°C, in EC2, BL1, and BL2. ^*^, ^**^ and ^***^ indicate significance levels of 0.05, 0.01 and 0.001, respectively.

We tested the effects on the K_ir_-current amplitude of 5–10 min applications of 5-HT (50 μM), dopamine (100 μM, + 1 mM ascorbic acid), quinpirole (D2 agonist, 30 μM), SKF 38393 (D1 agonist, 15 μM) noradrenaline (NA; 100 μM, + 1 mM ascorbic acid), phenylephrine (α1 agonist, 10 μM), clonidine (α2 agonist, 10 μM), histamine (10 μM), oxotremorine (muscarinic agonist, 10 μM) and baclofen (GABA_B_ agonist, 10 μM); the results, illustrated in Figure 9.

***NA***. The MOB receives a rich noradrenergic projection from the *locus cœruleus* (LC): approximately 40% of LC neurons (an estimated 400–600 out of 1600 cells) project to the rat OB (Shipley et al., 1985).

NA, acting via α1 receptors, has been reported to inhibit rectifying and non-rectifying leak potassium currents (Inokuchi et al., 1992; Vaughan et al., 1996; Hayar et al., 2001; Nai et al., 2010). We tested the NA (100 μM) on DA-PG cells in slice at 34°C observing a 24.6% reduction in the amplitude of the current activated by hyperpolarization: the current evoked at −100 mV decreased from −17.51 ± 1.62 pA/pF in control conditions to −13.20 ± 1.23 pA/pF in the presence of NA (*n* = 11; *p* < 0.001; Two-Way ANOVA; Figure 9A). Next, we tried to further characterize this effect identifying the subtype of α-receptor involved. Clonidine (α2 agonist, 10 μM) was ineffective (from −15.1 ± 1.4 pA/pF in control conditions to −14.1 ± 1.7 pA/pF with clonidine; *n* = 4; *p* = 0.4—Two-Way ANOVA; Figure 9A), whereas phenylephrine (α1 agonist, 10 μM) induced a 24.1% inhibition (from −12.9 ± 1.3 pA/pF in control conditions to −9.8 ± 0.92 pA/pF with phenylephrine; *n* = 8; *p* < 0.05—Two-Way ANOVA; Figure 9A), an inhibition almost identical to that of NA.

If the K_ir_ current deeply influences the resting potential, then we should expect that any modification of the amplitude of the current is paralleled by a variation of the membrane potential. In particular, in this case, a reduction of a hyperpolarizing current should be reflected in a depolarization of the cell in current-clamp experiments; this is exactly what can be observed (Figure 9B): phenylephrine 10 μM induces a depolarization of 8.1 ± 3.0 mV (*n* = 7; *p* < 0.05—*t*-test for paired data).

***ACh***. Cholinergic fibers from the horizontal limb of the diagonal band of Broca project to all bulbar layers, with the heaviest density occurring in the GL and EPL (Ichikawa and Hirata, 1986; Záborszky et al., 1986; Matsutani and Yamamoto, 2008); the OB itself appears to be devoid of intrinsic cholinergic neurons (Godfrey et al., 1980; Le Jeune and Jourdan, 1991; Butcher et al., 1992; Ichikawa et al., 1997), although this view has been more recently challenged (Krosnowski et al., 2012). The M2 agonist oxotremorine (10 μM) increases from −10.8 ± 1.1 pA/pF (CTL) to −12.4 ± 1.1 pA/pF the amplitude of the current (*n* = 13; *p* < 0.01; *t*-test for paired data), an effect which is paralleled by a 4.5 ± 0.8 mV hyperpolarization in current-clamp conditions (*n* = 9; *p* < 0.001; *t*-test for paired data).

***5-HT***. Serotonin (50 μM) induces a decrease of the K_ir_ current amplitude: at −100 mV the hyperpolarization-activated inward current is reduced from −22.8 ± 6.3 pA/pF (CTL) to −19.4 ± 5.3 pA/pF (*n* = 18, *p* < 0.01; Two-Way ANOVA), to which correspond a depolarization of 12.8 ± 3.2 mV (*n* = 8; *p* < 0.001; *t*-test for paired data) in current-clamp conditions.

***Histamine***. In voltage-clamp conditions, histamine (10 μM) induces a significant reduction of the K_ir_ current amplitude, which at −100 mV decreases from −19.0 ± 2.0 pA/pF (CTL) to −14.1 ± 1.8 pA/pF (test; *p* < 0.05, Two-Way ANOVA *n* = 8), an effect which is paralleled by a 10.9 ± 2.4 mV depolarization (*p* = 0.0013, *n* = 10; *t*-test for coupled data).

***DA***. The presence of autoreceptors is an hallmark of dopaminergic neurons, and therefore it was of interest to verify if their activation could modify the I_Kir_. Dopamine (100 μM) induces an increase of the K_ir_ current: in slice, at 34°C there is a nearly 17% increase of the current amplitude, from −16.9 ± 2.9 pA/pF (CTL) to −19.9 ± 2.3 pA/pF (current measured in response to a step to −100 mV; *n* = 5; *p* < 0.01; Two-Way ANOVA).

The effect is exactly mimicked by the D2 agonist quinpirole: 30 μM promotes an average increase 17%, from −15.5 ± 0.8 pA/pF (CTL) to −18.3 ± 1.2 pA/pF (current measured in response to a step to −100 mV; *n* = 9; *p* < 0.05; Two-Way ANOVA); on the contrary, the D1 agonist SKF 38393 (Sibley et al., 1982) remains ineffective (15 μM, *n* = 4; Figure 9A).

***GABA***. Kir3 channel family (GIRK) has been shown to be functionally regulated by GABA_B_ receptors in numerous systems (Sodickson and Bean, 1996; Lüscher et al., 1997; Tabata et al., 2005; David et al., 2006), including dopaminergic neurons (Lacey et al., 1988). We therefore tested the GABA_B_ agonist baclofen (Bowery et al., 1980) 10 μM on the K_ir_ current, without observing any effect (from −30.5 ± 4.6 pA/pF to −29.5 ± 5.0 pA/pF, *n* = 11; *p* > 0.5; Two-Way ANOVA; not shown).

## Discussion

Two hyperpolarization-activated currents with inward rectifying properties are present in TH-GFP+ neurons.

The first is an h-current (I_h_, or I_f_ in cardiac tissue), a mixed cation current with a reversal potential substantially positive to E_K_ (Hibino et al., 2010). I_h_ has a relatively slow activation kinetics, is insensitive to Ba^2+^, can be selectively blocked by drugs like ivabradine or ZD728, and does not show a voltage sensitivity dependent on [K^+^]_o_ (Biel et al., 2009). This current has been the object of a previous study (Pignatelli et al., 2013), and will not be further discussed here.

A second type of hyperpolarization-activated current is characterized by fast kinetics, is permeable primarily to K^+^, is blocked by extracellular Ba^2+^ and Cs^+^, has a voltage-dependence itself dependent on extracellular K^+^ concentration, and is identified as a classical inward rectifier potassium current (K_ir_). Sensitivity to Ba^2+^, insensitivity to selective h-current blockers, fast kinetics of activation and reversal potential, all suggest that the second hyperpolarization-activated current observed in TH-GFP+ neurons and described in this study belongs to this class.

Under physiological conditions, K_ir_ channels generate a large K^+^ conductance at potentials negative to *E*_K_, but permit a small current flow also at potentials positive to *E*_K_ (Hibino et al., 2010); as a result, the K_ir_ conductance has a tonic hyperpolarizing influence on the resting membrane potential (*V_rest_*), controlling excitability and affecting the repolarizing phase of the action potentials in excitable cells (Constanti and Galvan, 1983; Hume and Uehara, 1985; Day et al., 2005). In this study, we show that the K_ir_ current plays a key role in controlling *V_rest_* in DA-PG cells, neurons that -due to their strategic positioning at the entry of the bulbar circuitry and for direct connection with both the sensory input and projection neurons- are pivotal elements in the operation of glomerular circuits, and we show that the I_Kir_ in these cells is finely tuned by a variety of neurotransmitters.

### Which population of K_ir_ channels?

Of the seven main types of K_ir_ channels, at least two (KIR2.x and 3.x) are present in the MOB. Of the 2.x family, KIR2.1 is highly expressed in periglomerular cells (Prüss et al., 2005), as well as KIR2.2 (a.k.a. IRK2/KCNJ12; (Karschin et al., 1996); also KIR2.3 is weakly expressed in the glomerular layer (Inanobe et al., 2002; Allen Brain Atlas, 2013). Quinacrine, which differentially inhibits the K_ir_ channels (KIR2.3 > KIR2.1 » KIR6.2; (López-Izquierdo et al., 2011), suppresses a large (46%) fraction of hyperpolarization-activated inward current. However, the presence of KIR6.x (a.k.a. K_ATP_) channels can probably be excluded: these channels are thought to be octomers composed of four pore-forming K_ir_ subunits, and four auxiliary proteins, the sulfonylurea receptors (SURx) believed to be responsible for the channel (Hibino et al., 2010). SURx proteins are not detected in the MOB (Allen Brain Atlas, 2013), and therefore the more likely target of the action of quinacrine are 2.x K_ir_ channels, whose presence in the MOB would be confirmed by our data.

The presence of KIR3.x channels (G protein-coupled K_ir_, a.k.a. GIRK, channels) has been reported in the periglomerular layer of the MOB (Karschin et al., 1996); the sensitivity of a fraction of the hyperpolarization-activated inward current to tertiapin, a rather selective blocker of KIR3.1–3.4 channels (Jin and Lu, 1998; Kitamura et al., 2000; Ramu et al., 2008), would confirm this finding.

In conclusion, in control conditions, DA-PG cells display an inward rectifying current at hyperpolarizing potentials around E_K_. A first component is sustained by Ba^2+^-sensitive KIR2.x channels, which are constitutively active and which are well known to contribute to the resting K^+^ conductance in many cells (Hibino et al., 2010). On the other hand, this background activity could receive the contribution also of KIR3.x channels opening in response to G-protein activation by different neuromodulators, as discussed below.

### Pharmacology

Many neuromodulators such as NA, ACh, and 5-HT, play important functions in many sensory systems. As it occurs to other brain functions, sensory perception must be finely tuned according to task demands, qualities of sensory stimuli -such as strength or signal-to-noise ratio- and global physiological state. In this context, it is rather interesting that I_Kir_, a current playing such an important role in the resting membrane potential of cells strategically placed at the entry of the bulbar circuitry, can be modulated in both directions by a variety of neurotransmitters, all released in the region where DA-PG cells reside. The responses induced by neurotransmitters shown and discussed in this work are due to the direct activation of receptors on bulbar DA-PG cells, since all recordings were made in conditions of block of synaptic transmission.

#### NA

In this work, we show that in mouse DA-PG cells, NA and the α1 agonist phenylephrine significantly reduce the I_Kir_ and depolarize the cell.

Although the role of NA in olfactory function is one of the best studied in the OB (Trombley, 1994; Ciombor et al., 1999; Devore and Linster, 2012; Zimnik et al., 2013) to name a few, its effects at cellular, network and behavioral levels are somewhat discordant (Ennis and Hayar, 2008); it is worth noting that these inconsistencies have been ascribed at least in part to the absence of information pertaining to glomerular modulation by NA (Linster et al., 2011), a gap to fill which this work provides a first contribution.

The *locus cœruleus* neurons have been proposed to influence external signal processing so that salient stimuli are enhanced and the activity more related to tonic, vegetative functions is suppressed (Aston-Jones et al., 2000). In the OB, the NA release in response to LC activation should bring the DA-PG neurons to a more excited state for the inhibition of the K_ir_ conductance. This, for the known presynaptic inhibitory effect of DA (Koster et al., 1999; Ennis et al., 2001), would reduce the release of glutamate from the olfactory nerve terminals, improving the signal-to-noise ratio of the information coming from the olfactory epithelium, an effect that would be in line with the postulated general role of the LC on sensory stimuli perception (Aston-Jones et al., 2000).

An additional role of NA on dopaminergic cells might be of some interest: a significant fraction of the interneurons added in adulthood to the glomerular layer of the MOB are dopaminergic (Pignatelli et al., 2009), and noradrenaline signaling enhances newborn cell survival (Bauer et al., 2003; Bovetti et al., 2011).

#### ACh

In the MOB, ACh, acting on both nicotinic and muscarinic receptors, has a complex effect (for a review see Devore and Linster, 2012). Overall, the resulting effect of cholinergic modulation is excitatory (Elaagouby and Gervais, 1992) and the multiple action of ACh seems to be orchestrated toward an enhancement of specificity and temporal precision of mitral cell responses to odors (Elaagouby and Gervais, 1992; Mandairon et al., 2006; Tsuno et al., 2008; D'Souza and Vijayaraghavan, 2012).

In this work we show that in DA-PG cells the activation of M2 muscarinic receptors increases the amplitude of a K_ir_ current, an effect which is paralleled by a 4.5 mV hyperpolarization in current-clamp conditions. A similar effect has been reported in a variety of preparations, ranging from mammal atrial myocytes (Sakmann et al., 1983), to thalamic reticular neurons (Mccormick and Prince, 1986), spinal motoneurons (Chevallier et al., 2008), interneurons of striatum (Calabresi et al., 1998), neocortex (Xiang et al., 1998), and hippocampus (McQuiston and Madison, 1999).

M2-type muscarinic receptors are described in the glomerular layer associated to PG-DA neurons (Crespo et al., 2000; Allen Brain Atlas, 2013). In a previous paper (Pignatelli and Belluzzi, 2008), we showed that the activation of M2 metabotropic cholinergic receptor in PG-DA neurons induced a hyperpolarization mediated by a K-conductance, which in the present work is now identified as a K_ir_.

#### 5-HT

Projections from the dorsal and median raphe nuclei -one of the most prominent neuromodulatory systems in the brain- provide a dense serotonergic innervation of the MOB, and in particular of the glomerular region (Araneda et al., 1980; McLean and Shipley, 1987). Earlier studies have shown that 5-HT2 receptor mRNA and protein are heavily expressed in the glomerular layer (Morilak et al., 1993) and activation of 5-HT(2C) receptors mediates excitation in about one third of glomerular neurons, not better identified (Hardy et al., 2005); this study further develops this observations showing that serotonin produces excitatory modulation of DA-PG cells by reducing the I_Kir_ amplitude, thereby depolarizing the cell for 12.8 ± 3.2 mV.

A similar action on I_Kir_ is described also in rat motoneurons (Kjaerulff and Kiehn, 2001).

#### Histamine

The MOB receives histaminergic inputs primarily from the caudal tuberal and postmammillary magnocellular hypothalamus (Auvinen and Panula, 1988; Panula et al., 1989) via the olfactory peduncle (Brunjes, 2013), and previous studies have shown that in an unidentified fraction of periglomerular cells, H1-receptor activation causes a block of a potassium current (Jahn et al., 1995). Here we show that the I_Kir_ in DA-PG cells is reduced and that this action results in a depolarization of the cells.

#### DA

Dopamine (100 μM) induces an increase of the K_ir_ current via D2R; the D2R agonist quinpirole (30 μM) perfectly replicates the effect of dopamine. Further experiments using receptor protection with D1R selective antagonists might exclude more definitively a contribution from D1R, although a direct activation of D1R with SKF 38393 (15 μM) was completely ineffective. We did not investigate the pathways involved.

## Concluding observations

In the present study we have shown that (i) DA-PG cells contain a large inward rectifier current whose block produces significant depolarizations, nominating this conductance as one of the main players controlling the resting membrane potential (and consequently excitability) in these cells, and that (ii) this current is subject to a complex modulation.

Bulbar DA-PG cells, the largest and one of the most conserved populations of DA neurons in the CNS, are pivotal neurons in the operation of glomerular circuits, the site where odor information is initially processed. It is therefore of some interest that their excitability is profoundly dependent upon the K_ir_ current, and that this -in turn- is target of numerous neurotransmitters that can finely modulate its amplitude, a process that ultimately impacts all subsequent odor processing in the olfactory system.

In this context, it is increasingly evident that in the bulb as a whole there is an enormous and still largely hidden layer of “molecular computation” (Bhalla, 2014), which multiplies tremendously the degrees of freedom of the bulbar network in signal processing.

### Conflict of interest statement

The authors declare that the research was conducted in the absence of any commercial or financial relationships that could be construed as a potential conflict of interest.
